# Atomic Oxygen-Resistant Polyimide Composite Films Containing Nanocaged Polyhedral Oligomeric Silsesquioxane Components in Matrix and Fillers

**DOI:** 10.3390/nano11010141

**Published:** 2021-01-08

**Authors:** Yan Zhang, Hao Wu, Yi-dan Guo, Yan-bin Yang, Qiang Yu, Jin-gang Liu, Bo-han Wu, Feng-zhu Lv

**Affiliations:** 1 Beijing Key Laboratory of Materials Utilization of Nonmetallic Minerals and Solid Wastes, National Laboratory of Mineral Materials, School of Materials Science and Technology, China University of Geosciences, Beijing 100083, China; 3003200016@cugb.edu.cn (Y.Z.); 2003200021@cugb.edu.cn (H.W.); guoyidan163@163.com (Y.-d.G.); 2 Space Materials and Structure Protection Division, Beijing Institute of Spacecraft Environment Engineering, Beijing 100094, China; yanbinyang_cast@163.com (Y.-b.Y.); 13426373533@139.com (Q.Y.)

**Keywords:** polyimide film, polyhedral oligomeric silsesquioxane, atomic oxygen, thermal properties, self-healing

## Abstract

For the development of spacecraft with long-servicing life in low earth orbit (LEO), high-temperature resistant polymer films with long-term atomic oxygen (AO) resistant features are highly desired. The relatively poor AO resistance of standard polyimide (PI) films greatly limited their applications in LEO spacecraft. In this work, we successfully prepared a series of novel AO resistant PI composite films containing nanocaged polyhedral oligomeric silsesquioxane (POSS) components in both the PI matrix and the fillers. The POSS-containing PI matrix film was prepared from a POSS-substituted aromatic diamine, *N*-[(heptaisobutyl-POSS)propyl]-3,5-diaminobenzamide (DABA-POSS) and a common aromatic diamine, 4,4′-oxydianline (ODA) and the aromatic dianhydride, pyromellitic dianhydride (PMDA) by a two-step thermal imidization procedure. The POSS-containing filler, trisilanolphenyl POSS (TSP-POSS) was added with the fixed proportion of 20 wt% in the final films. Incorporation of TSP-POSS additive apparently improved the thermal stability, but decreased the high-temperature dimensional stable nature of the PI composite films. The 5% weight loss temperature (*T*_5%_) of POSS-PI-20 with 20 wt% of DABA-POSS is 564 °C, and its coefficient of linear thermal expansion (CTE) is 81.0 × 10^−6^/K. The former is 16 °C lower and the latter was 20.0 × 10^−6^/K higher than those of the POSS-PI-10 film (*T*_5%_ = 580 °C, CTE = 61.0 × 10^−6^/K), respectively. POSS components endowed the PI composite films excellent AO resistance and self-healing characteristics in AO environments. POSS-PI-30 exhibits the lowest AO erosion yield (*E*_s_) of 1.64 × 10^−26^ cm^3^/atom under AO exposure with a flux of 2.51 × 10^21^ atoms/cm^2^, which is more than two orders of magnitude lower than the referenced PI (PMDA-ODA) film. Inert silica or silicate passivation layers were detected on the surface of the PI composite films exposed to AO.

## 1. Introduction

Research on the improvement of the atomic oxygen (AO)-resistant properties of standard polyimide (PI) films have become one of the most important topics in the development of high-performance polymer materials for low earth orbit (LEO) spacecraft [1,2,3]. The standard PI films, such as poly(pyromellitic dianhydride-4,4′-oxydianiline) (PI_PMDA-ODA_, trademark: Kapton^®^, Dupont, DE, USA), poly(3,3′,4,4′-biphenyltetracarboxylic dianhydride-1,4-phenylenediamine) (PI_BPDA-PDA_, trademark: Upilex-S^®^, Ube, Japan) have been widely used in spacecraft due to their excellent combined properties, including good thermal resistance, excellent radiation resistance, good mechanical properties, and so on [1]. However, they are usually subject to AO erosion with an erosion yield at the level of 10^−24^ cm^3^/atom in LEO space environments [4,5,6]. This means that the PI film designed and served as the protecting layers for LEO spacecraft themselves might be eroded by AO exposure first, thereby losing the protecting functions. Even if the PI films are protected by AO-resistant surface coatings or fillers, such as silica, titania, alumina, germanium, and so on, the highly active AO might destroy the PI film matrixes via an undercutting route [7,8].

After several decades of basic and applied studies for the AO erosion mechanisms of standard PI films, there have been establishing some effective procedures for enhancing the AO-resistant abilities of the PI films. These procedures can roughly be classified as the passive and active ones. Both procedures obey the same mechanism and all achieve the AO protection via the formation of inorganic passivation layers [9,10]. The difference lies in the pathways achieving the purpose. The former usually provides the AO protections directly via applying surface coatings or external fillers [11,12,13]. The latter usually indirectly achieve the active protection via incorporation of specific elements, such as silicon or phosphorus, which could in-situ form the inorganic oxides passivation layer when being exposed in an AO environment [14,15,16]. Both procedures have advantages and disadvantages. The passive procedures are usually cost-effective and can usually achieve excellent AO protection to the PI films in short-term LEO exploration. However, the procedure usually suffers from the AO undercutting due to the unavoidable defects in the coatings and uniformity in the dispersion of the fillers [17,18]. The active procedures usually require the design and development of new functional monomers, which makes them cost-sensitive. In addition, the incorporation of new monomers might make the PI films inherent thermal and mechanical properties deteriorate. At last, in order to form the effective passivation layers, considerable amounts of the polymer matrix have to be consumed [19,20].

Considering the individual disadvantages for these two procedures, a combination of the passive and active pathways was proposed in recent years. In our previous work, the AO-resistant POSS fillers were used in combination with the AO-resistant phosphorus-containing matrix, so that the obtained composite films had excellent AO resistance. Due to the synergistic effect of phosphorus and silicon elements, the AO erosion yield of the derived composite films was as low as 3.1 × 10^−26^ cm^3^/atom (Fluence: 4.0 × 10^20^ atoms/cm^2^) [21]. The lowest erosion yield was only 2.2% of that of referenced Kapton^®^ films. Song, et al. also reported the synergistic improvement of AO resistance of PI films by introduction of both phosphorus and silicon elements [22]. Although the combined procedures provided excellent AO resistance for the PI films, the compatibility between the PI matrix and the fillers has to be addressed so as to achieve the best protective efficiency.

In recent years, polyhedral oligomeric silsesquioxane (POSS) have been paid increasing attention as a promising component to enhance the AO resistant properties of the PI films due to the great potential ability to form silicon-containing passivation layers when reacted with AO [23]. Various POSS-substituted PI films, either in the positions of main chain or side chain of the PI molecular chains have been reported in the literature [24,25,26]. In 2012, Minton and coworkers systemically reported the research and development of main-chain POSS-substituted Kapton^®^ PI film (MC-POSS-Kapton) and side-chain substituted one (SC-POSS-Kapton) [24]. Both POSS-containing PI films have been investigated as high-performance AO-resistant candidates in real LEO space environments and the results showed that it was a promising and practical pathway to enhance the AO-resistant properties of the standard Kapton^®^ films by combining POSS units into the PI films via copolymerization. Especially, the SC-POSS-Kapton^®^ film exhibited relatively lower cost and comparable AO resistance compared with the main chain one. Thus, SC-POSS-Kapton^®^ showed great application future in long-term LEO exploration. In addition, POSS compounds have also been used as the fillers to develop PI composite films with improved AO resistance [27,28,29]. In 2016, Qian et al. reported the AO behaviors of PI films derived from the Kapton^®^ matrix and the trisilanolphenyl POSS (TSP-POSS) additives [27]. TSP-POSS endowed the good AO resistance of the composite films while maintaining the inherent thermal and mechanical properties of the Kapton^®^ matrix film. The AO erosion yields of TSP-POSS/Kapton composite films are comparable to those of the MC-POSS-Kapton and SC-POSS-Kapton films.

**Figure 1:**
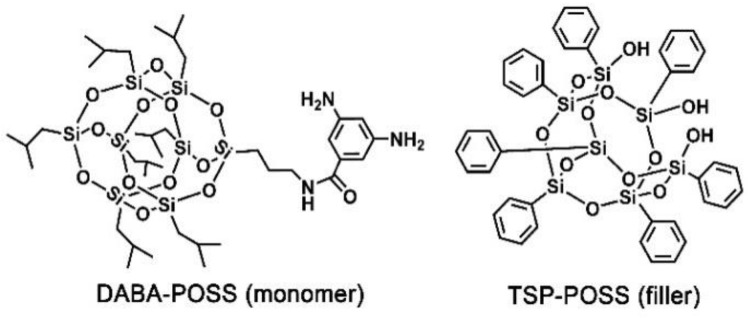
Nanocaged polyhedral oligomeric silsesquioxane (POSS) units used in the current work.

In our previous work, the effects of the side-chain substituted POSS units on the optical, thermal, mechanical, and AO erosion behaviors of PI (PMDA-ODA) films were systemically reported [30]. In the current work, the POSS units were endeavored to incorporate into the PI (PMDA-ODA) films via the combination of copolymerization and physical blending procedures. The copolymerization was achieved by the use of the diamine monomer, DABA-POSS, and the blending was performed by the use of TSP-POSS additives, whose molecular structures are shown in Figure 1. Effects of the side-chain POSS and the external POSS components on the physical and chemical properties, especially thermal and AO erosion behaviors were investigated in detail.

## 2. Materials and Methods

### 2.1. Materials

TSP-POSS was obtained from Hybrid Plastics, Co. Ltd., (Hattiesburg, MS, USA). DABA-POSS was prepared according to our previous work [30]. Pyromellitic dianhydride (PMDA) and 4,4′-Oxydianline (ODA) were purchased from Tokyo Chemical Industry (TCI) Co., Ltd., Tokyo, Japan. PMDA was treated under vacuum at 180° for 10 h to remove the water absorbed prior to use, ODA was used directly. Ultra-dry solvents with the water contents below 200 ppm, including *N*-methyl-2-pyrrolidinone (NMP), *N,N*-dimethylacetamide (DMAc), *N*,*N*-dimethylformamide (DMF) were purchased from Sinopharm Chem Reagent Co. Ltd. (Shanghai, China) without purification.

### 2.2. Measurements

The attenuated total reflectance Fourier transform infrared (ATR-FTIR) spectra of PI composite films were obtained using a Bruker Tensor-27 FT-IR spectrometer (Ettlingen, Germany) with the scan range of wavenumber from 4000 to 400 cm^−1^. The wide-angle X-ray diffraction (XRD) patterns of PI composite films were determined with Rigaku D/max-2500 X-ray diffractometer (Tokyo, Japan) using Cu-Kα1 radiation at room temperature between 3 °C and 80 °C. X-ray photoelectron spectroscopy (XPS) data were measured with ESCALab220i-XL electron spectrometer (Thermo Fisher Scientific, Waltham, MA, USA). XPS spectra were obtained using MgKα X-ray source with a power of 300 W. The base pressure of vacuum chamber was set at 3 × 10^−9^ mbar. The binding energies were 284.8 eV derived from the adventitious C1s as a reference. Field emission scanning electron microscopy (FE-SEM) was carried out using a Technex Lab Tiny-SEM 1540 (Tokyo, Japan) with an accelerating voltage of 15 KV for imaging. Pt/Pd was sputtered on each film before the SEM measurements. The atomic force microscopy (AFM) images of PI composite films were measured in tapping mode on a Bruker Multimode 8 AFM microscope (Santa Barbara, CA, USA).

For the transparency measurements, ultraviolet-visible (UV-Vis) spectra of PI composite films were recorded on a Hitachi U-3210 spectrophotometer (Tokyo, Japan) at room temperature. PI samples were dried at 100 °C for 1 h to remove any absorbed moisture prior to tests. Yellow index (YI) and haze values of the PI films (thickness: 20 µm) were measured using an X-rite Ci7800 spectrophotometer (Grand Rapids, MI, USA).

Thermogravimetric analysis (TGA) was performed with a Perkin-Elmer TGA4000 thermal analysis system (Waltham, MA, USA). The thermal scanning mode ranges from 50 to 750 °C at a heating rate of 10 K/min in nitrogen atmosphere with a gas flow of 20 mL/min. Similarly, thermo-mechanical analysis (TMA) was recorded on a TA-Q 400 thermal analysis system (New Castle, DL, USA). The thermal scanning mode ranges from 50 to 400 °C at a heating rate of 10 K/min in nitrogen atmosphere. The size of the film samples was 10 × 5 × 0.025 mm^3^. The coefficients of linear thermal expansion (CTE) values of composite films were recorded in the range of 50–200 °C.

The AO erosion behaviors of PI composite films were tested in a ground-based AO effect simulation facility [30]. The AO fluence was measured after exposure by the etch depth of Kapton^®^ reference sample, whose erosion yield (*E*_Kapton_) was 3.0 × 10^−24^ cm^3^/atom. The fluence could be calculated from the etch depth by the following Equation (1):

(1) $$F=\frac{\Delta H_{Kapton}}{E_{Kapton}}$$

where, *F* = AO fluence (atoms/cm^2^); Δ*H*_Kapton_ = erosion depth of the Kapton^®^ reference sample (cm); *E*_Kapton_ = erosion yield of Kapton^®^ reference sample (3.0 × 10^−24^ cm^3^/atom).

According to the equation above, the fluence of AO in this experiment is calculated to be 2.51 × 10^21^ atoms/cm^2^. In order to study the etching depth, the samples were covered with stainless steel meshes to produce the etched and pristine areas, which were used to measure the step height using a profilometer. The erosion yield of the sample, *E*_s_, is calculated through the following Equation (2) [27]:

(2) $$E_{s}=\frac{\Delta H_{s}}{\Delta H_{Kapton}}E_{Kapton}$$

where, *E*_s_ = erosion yield of the sample (cm^3^/atom); Δ*H*_s_ = erosion depth of the sample (cm).

### 2.3. Synthesis of PAA Varnishes and Preparation of PI Composite Film

The preparation of poly(amic acid) (PAA) composite solutions was achieved by the combination of copolymerization and physical blending of POSS-containing components, which could be illustrated by the synthesis of POSS-PAA-30 containing 30 wt% of DABA-POSS and 20 wt% of TSP-POSS. The experimental device is equipped with a mechanical stirrer, an ice-cold bath, and a 500-mL three-necked round-bottom flask with nitrogen inlet. First, ODA (15.35 g, 76.67 mmol), DABA-POSS (15.15 g, 15.02 mmol), TSP-POSS (12.63 g) and ultra-dry DMAc solvent (182.5 g) were added into the flask. A clear solution was obtained after stirring at 5–10 °C for 2 h under nitrogen flow. Then, PMDA (20.00 g, 91.69 mmol) and the additional DMAc (70.0 g) were added to the reaction mixture obtained above. The solid content of the reaction mixture was adjusted to be 20 wt% by this procedure. The cold bath was removed and the reaction mixture was stirred at room temperature for another 22 h. The deep-brown viscous solution obtained was filtered through a 0.45-µm polytetrafluoroethylene (PTFE) syringe filter. Then the prepared POSS-PAA-30 solution was stored in a glass bottle at −18 °C before use.

The degassed POSS-PAA-30 solution was warmed to room temperature before use, and then blade-coated onto a clean glass substrate. The thickness of the wet PAA films was controlled by the height of the slit. The glass substrates were then placed in an oven with flowing nitrogen gas. The POSS-PAA-30 was gradually thermally imidized with the following curing procedure: 80 °C/3 h, 120 °C/1 h, 150 °C/1 h, 180 °C/1 h, 250 °C/1 h, 300 °C/1 h, and 350 °C/0.5 h. Then, the glass substrate was immersed in warm deionized water to obtain the free-standing POSS-PI-30 film.

The other PAA solutions and the corresponding PI films, including POSS-PI-10, POSS-PI-15, POSS-PI-20, and POSS-PI-25 were prepared according to a similar procedure. The difference was the contents of DABA-POSS were different. The pristine PI (PMDA-ODA) film was also prepared in a similar way.

## 3. Results and Discussion

### 3.1. PI Composite Films Preparation

**Figure 2:**
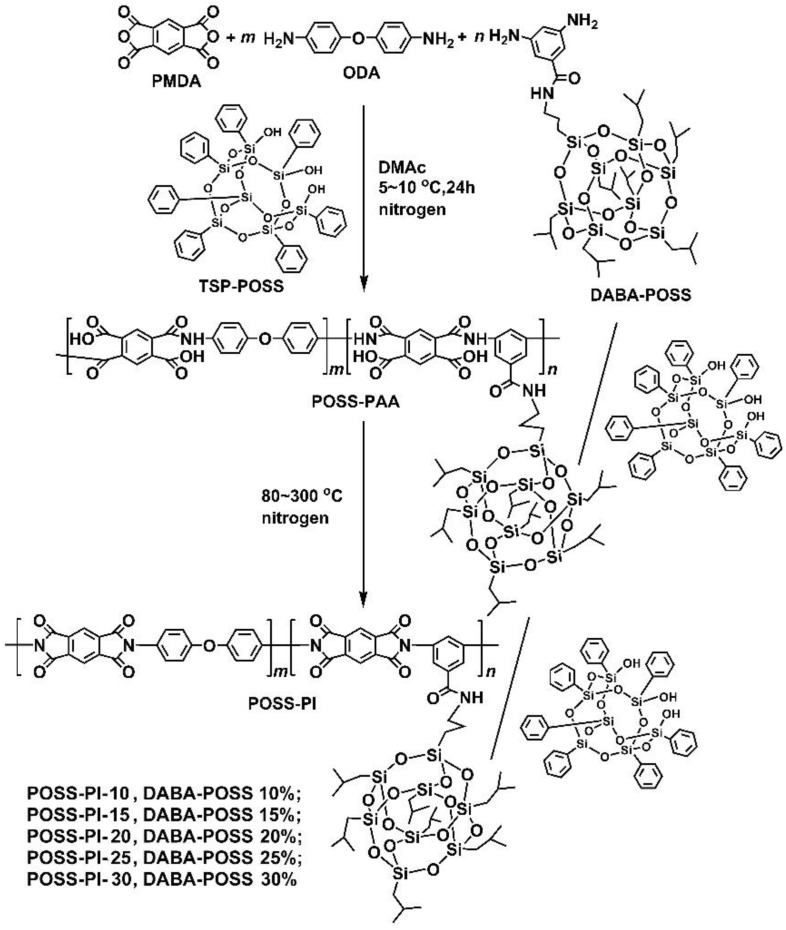
Synthesis of POSS-poly(amic acid) (PAA) varnishes and the derived POSS-polyimide (PI) composite films.

**Figure 3:**
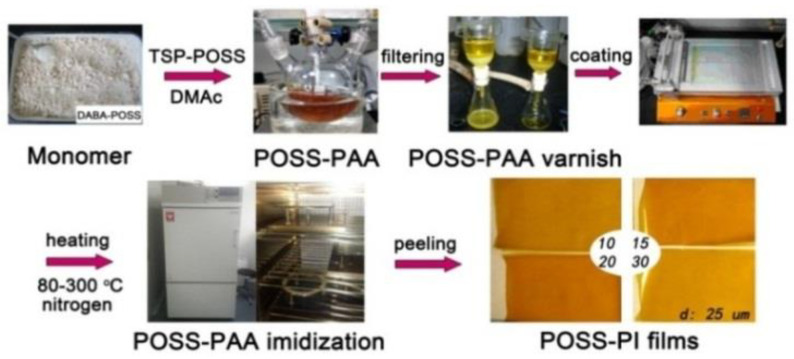
Preparation procedure of POSS-PI films.

A serious of POSS-PAA composite varnishes were first prepared by the combination of copolymerization and physical blending procedures according to the chemical reaction equation shown in Figure 2 and the illustrated procedure shown in Figure 3. The detailed synthesis formulations are listed in Table 1. The weight proportion of the TSP-POSS additives in the final PI films was controlled to be 20 wt%, while the weight proportion of the DABA-POSS was set to from 10 wt% to 30 wt%. The selected amount of 20 wt% for the TSP-POSS filler is based on our previous work [21], at which the excellent combined properties for the PI composite films were obtained. DABA-POSS exhibited good polymerization reactivity and afforded POSS-PAA solutions high viscosities, indicating the high molecular weights of the polymers. TSP-POSS additive showed good compatibility with the PAA matrix, as could be evidenced by the homogeneous and transparent appearance of the obtained PAA composite solutions, in which no phase separation and gelling occurred even being stored at −18 °C for several months. The corresponding PI composite films were obtained by thermally imidized the precursors POSS-PAA from 80 to 350 °C in nitrogen. The fingernail-creasable PI composite films showed good flexibility and toughness.

**Figure 4:**
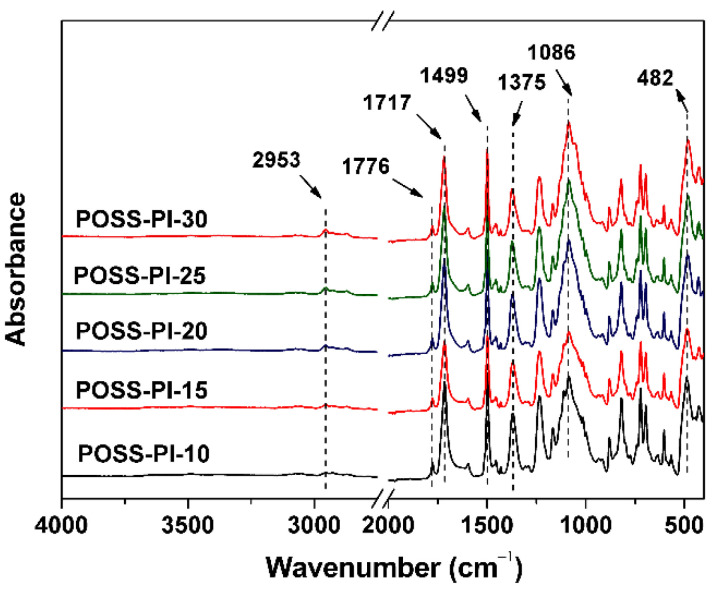
Attenuated total reflectance Fourier transform infrared (ATR-FTIR) spectra of POSS-PI composite films.

The successful incorporation of POSS units into the composite films, on one hand could be directly proven by the structure characterization, such as ATR-FTIR and XPS measurements, and on the other hand could be indirectly reflected by the XRD and optical properties evaluation. Figure 4 shows the ATR-FTIR spectra and the typical absorptions of the POSS-PI composite films. First, the asymmetrical stretching vibration and symmetric stretching vibration are at 1776 cm^−1^ and 1717 cm^−1^, respectively, and the stretching vibration of C-N bonds are at 1375 cm^−1^. These are the characteristic absorptions of imide rings. The characteristic absorptions of C=C bonds at 1499 cm^−1^ in phenyl units were observed in all of the PI systems in the spectra. Moreover, the characteristic absorptions of Si-O-Si in both of the DABA-POSS and the TSP-POSS were all detected at 1086 cm^−1^. In addition, the peaks at 2953 cm^−1^ could be assigned to the absorptions of saturated C-H bonds in isobutyl groups in DABA-POSS units. The absorptions of Si-OH in the TSP-POSS filler at the wavenumber around 3300–3500 cm^−1^ were not observed in the spectra, which are in consistence with the other PI/TSP-POSS system [31]. This might be due to the low contents of the Si-OH units in the PI composite films.

**Figure 5:**
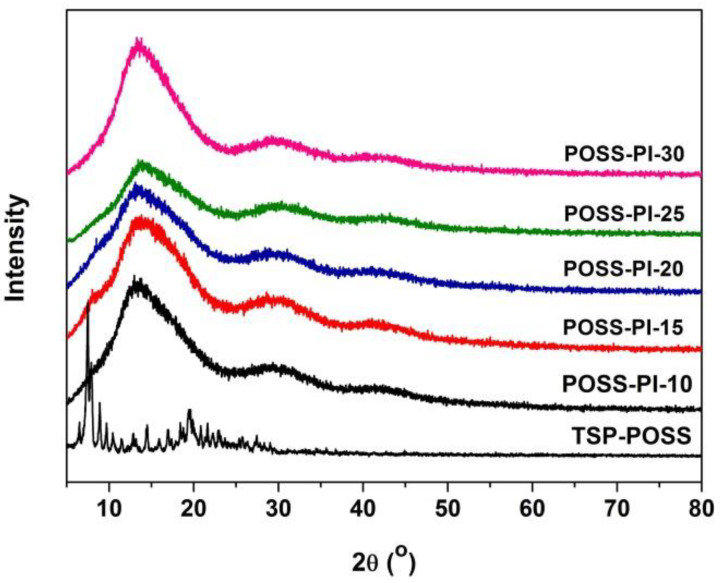
XRD patterns of POSS-PI composite films.

The XRD patterns of the POSS-PI composite film were shown in Figure 5. The sharp reflection of the TSP-POSS compound disappeared in the spectra of the composite films, indicating that the POSS-additives have good compatibility with the PI-matrix. This might be due to the similarity of the chemical structures for the POSS components. In addition, the bulky POSS units apparently decreased the crystallinity of the PI molecular chains, thereby giving the films an amorphous property.

**Figure 6:**
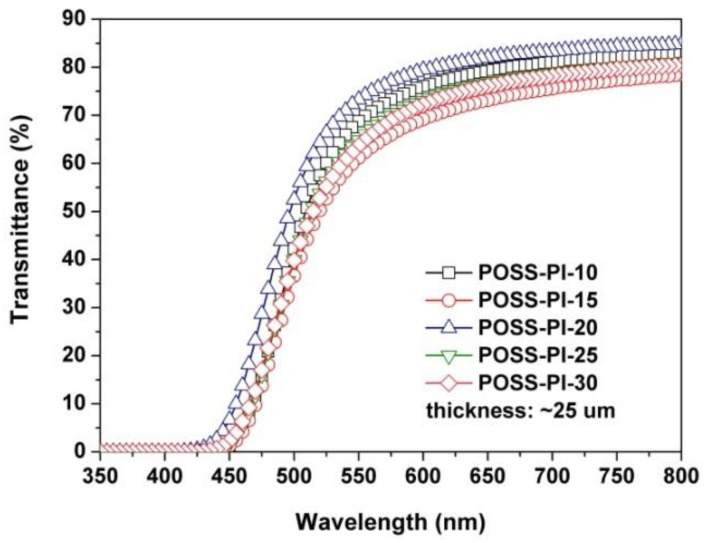
UV-Vis spectra of POSS-PI composite films.

**Figure 7:**
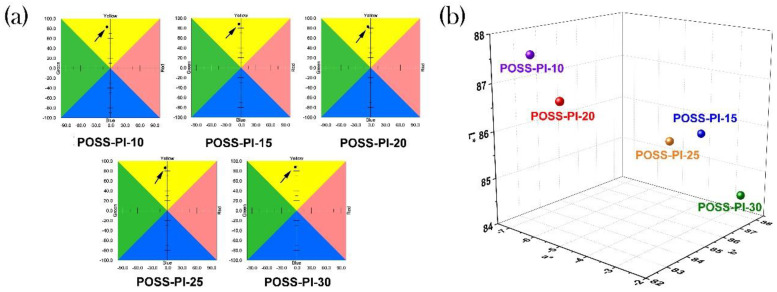
2D and 3D map for CIE Lab parameters of POSS-PI composite films. (**a**) 2D map; (**b**) 3D map.

The good miscibility of the additives with the DABA-POSS-PI matrix could be further revealed by the optical characteristics of the composite films. The optical data are tabulated in Table 2. Figure 6 and Figure 7 present the UV-Vis spectra and the CIE Lab optical parameters of the PI composite films, respectively. As can be seen from Figure 5 and Table 2, the PI composite films presented similar optical transparency with the transmittance values of 61.2–73.3% at the wavelength of 550 nm and UV cutoff wavelengths (λ_cut_) between 411 nm and 431 nm. Incorporation of the TSP-POSS additives into the films slightly increased the yellow indices (b*) and haze values of the composite films. Obviously, the haze values of the PI composite films increased from 7.19% to 15.82% when the contents of DABA-POSS increased from 10 to 30 wt% in the films. Furthermore, the haze values of the pristine DABA-POSS-PI films and the composites films are compared in Table 2. It can be clearly deduced from the data that addition of TSP-POSS deteriorated the haze values of the composite films when the contents of DABA-POSS were lower than 20 wt%. However, the PI composite films gradually exhibited lower haze values at higher DABA-POSS contents. POSS-PI-30 with the DABA-POSS content of 30 wt% and the TSP-POSS content of 20 wt% showed a haze value of 15.82%, which is about half of the analogous PI film without TSP-POSS additives (haze = 31.70%). This might be attributed to the formation of continuous POSS components in the composite films at higher loadings, which efficiently reducing the light scattering caused by the dispersed POSS components at lower contents.

### 3.2. Thermal Properties

**Figure 8:**
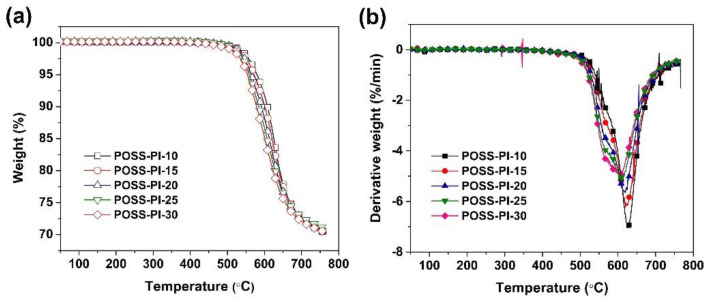
Thermal decomposition of POSS-PI composite films at a heating rate of 10 K/min under nitrogen flow. (**a**) TGA; (**b**) DTG.

In order to evaluate the thermal stability of the PI composite films, TGA and TMA measurements was used, and the data obtained are summarized in Table 3. The TGA and corresponding derivative TGA (DTG) plots of the PI films are shown in Figure 8. The POSS-PI composite films displayed good thermal stability before 450 °C in nitrogen, and then the films started decomposing and maintained nearly 60 wt% of their original weights at 750 °C. The 5% weight loss temperatures (*T*_5%_) of the composite films decreased with the increasing contents of DABA-POSS in the polymers. Basically, the *T*_5%_ values of the film decrease by 5–8 °C for every 5% increase of the DABA-POSS contents in the film. What’s more, addition of TSP-POSS apparently improved the thermal decomposition temperatures of the PI composite films. The *T*_5%_ values of the composite films were about 40 °C higher than those of the pristine films without TSP-POSS additives. For example, POSS-PI-30 film showed a *T*_5%_ value of 554 °C, which was 42 °C higher than that of the PI-30 film without TSP-POSS [30]. Higher silicon contents in the PI composite films afforded much higher char yield of the films at high temperatures. The residual weight ratios at 750 °C (*R*_w750_) values of the composite films were all higher than 70.0% in nitrogen, while the un-filled films showed the *R*_w750_ values below 60.0%. The increase of the thermal stability of the composite films might be owing to the enhanced interactions and miscibility between the TSP-POSS additives and the POSS-containing PI matrix. According to the DTG plots in Figure 8b, the maximum decompose temperatures (*T*_max_) for the PI composite films were all higher than 600 °C and also decreased with the increase of the DABA-POSS contents in the composite films.

**Figure 9:**
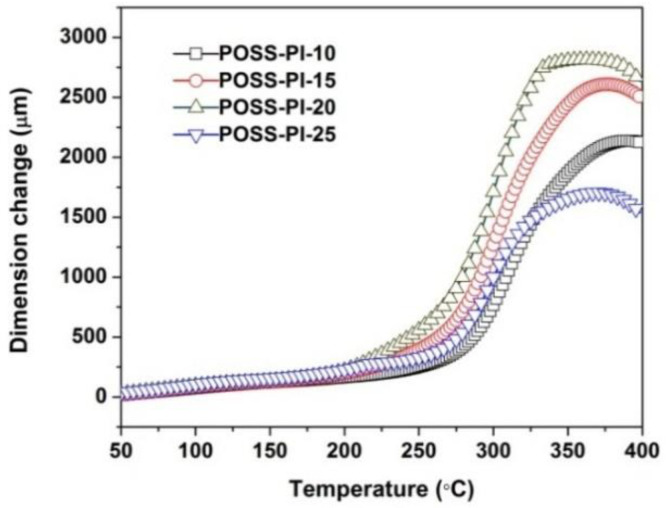
TMA curves of POSS-PI composite films under nitrogen flow.

In our previous work, it has been found that the introduction of side-chain-substituted POSS units deteriorated the high-temperature dimensional stability of the derived PI films because of the easy movement of the latent POSS groups at elevated temperatures [30]. In the current work, the addition of TSP-POSS additives further increased the CTE values of the derived PI composite films, which could be deduced from the TMA curves of the films shown in Figure 9. For example, the CTE value of POSS-PI-20 film in the temperature range of 50–200 °C is 81.0 × 10^−6^/K, which was obviously higher than the CTE of the analogous PI film without TSP-POSS additives (CTE = 56.1 × 10^−6^/K). It is possible that the TSP-POSS additive act as a “plasticizer”, which made the matrix easy to move at elevated temperatures.

### 3.3. AO Erosion Properties

**Figure 10:**
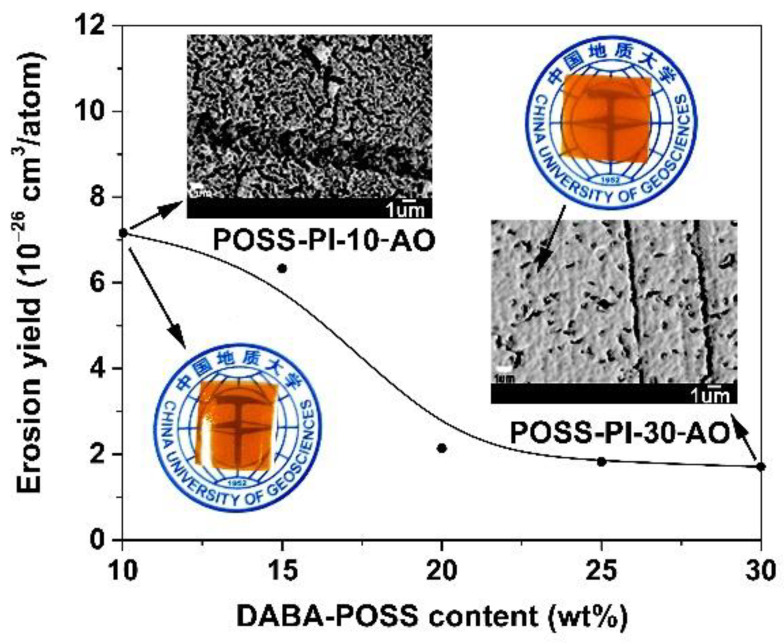
AO erosion yields as a function of DABA-POSS contents for the POSS-PI composite films (Insert: Appearance and field emission scanning electron microscopy (FE-SEM) images of POSS-PI-10-AO and POSS-PI-30-AO exposure to 2.51 × 10^21^ atom/cm^2^ AO attack, respectively).

**Figure 11:**
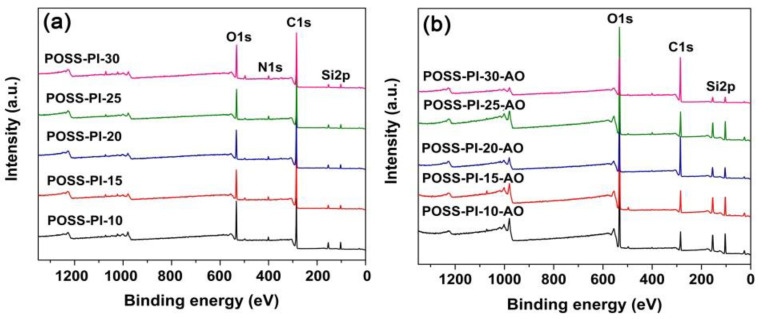
X-ray photoelectron spectroscopy (XPS) spectra of POSS-PI composite films. (**a**) Before AO exposure; (**b**) After AO exposure.

The ground-simulated AO facility was used to investigate the AO erosion behaviours of the composite films. And the experimental erosion yields (*E*_s_) of the PI films were tabulated in Table 4. The fluence of atomic oxygen is 2.51 × 10^21^ atoms/cm^2^. Figure 10 illustrates the *E*_s_ values of the POSS-PI films together with the representative appearances and SEM images of the POSS-PI-10 and POSS-PI-30 films after AO erosion, respectively. It can be clearly observed that as the POSS contents in the composite films are higher, the *E*_s_ values are lower. The *E*_s_ value of the POSS-PI-30 film was 1.64 × 10^−26^ cm^3^/atom, which was nearly an order of magnitude lower than the *E*_s_ value of the PI sample without TSP-POSS filler (*E*_s_ = 11.1 × 10^−26^ cm^3^/atom) and was only 0.55% of the referenced Kapton^®^ film (*E*_s_ = 300 × 10^−26^ cm^3^/atom). The lowest *E*_s_ value of the novel PI composite films developed in the current work is more than two orders of magnitude lower than that of the standard Kapton^®^ film. The big difference in the *E*_s_ values of these two series of PI films is mainly due to the synergistic effects of the nanocaged POSS units in both matrix and the additives for the newly developed PI composite films. The POSS units effectively decreased the AO erosion of the PI composite films. In addition, AO exposure deteriorated the optical transparency of the PI composite films, and a relatively dense layer could be observed on the surface of the POSS-PI-30 film through SEM detection. The surface elemental compositions of the PI composite films before and after AO exposure were then detected by XPS. The plots were shown in Figure 11, and the corresponding data were listed in Table 4. The elemental proportions of the silicon (Si) and oxygen (O) on the surface of the films increased significantly after AO exposure; however, those of the carbon (C) and nitrogen (N) decreased. By comparing the shifts of the binding energies for the Si2p and O1s components, SiO_x_ or silicate layers could be confirmed to form on the surface of the AO-eroded PI samples. Undoubtedly, these inert passivation layers provided efficient protection for the under-layer films.

**Figure 12:**
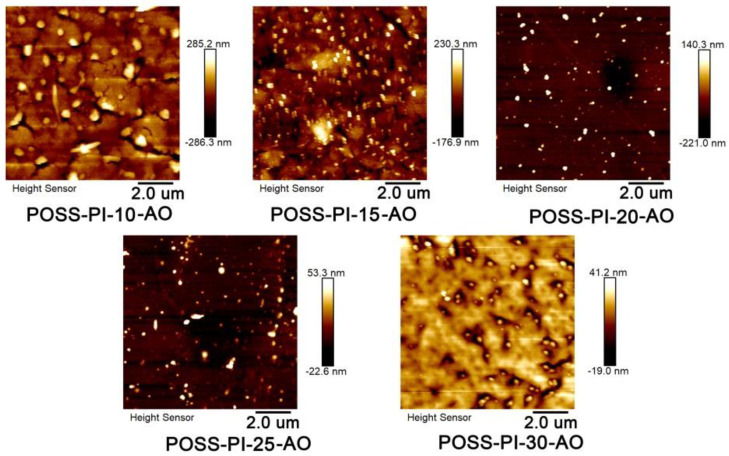
Atomic force microscopy (AFM) patterns of POSS-PI composite films after AO exposure (Fluence: 2.51 × 10^21^ atom/cm^2^).

The formation of the passivation layers caused by AO exposure could also be verified by inspecting the surface roughness of the AO-attacked surface of the composite films. Figure 12 showed the AFM images of the AO-eroded POSS-PI composite films in a 2D height scan on an area of 10 µm × 10 µm. AO exposure obviously increased the surface roughness of the films, as indicated by the large peak-to-valley depth (*R*_t_). Basically, the surface roughness caused by the AO exposure decreased gradually with the increasing contents of POSS components in the films due to the formation of compact passivation layer. For instance, the *R*_t_ values of the composite films decreased from the initial value of −286.3–285.2 nm of POSS-PI-10-AO to −19.0–41.2 nm of POSS-PI-30-AO. This result indicates that the distribution of passivation layer onto the surface of the composite films progressively became dense and compact, thus providing much efficient protection for the underlying films and apparently reducing the AO erosion yield of the composite films.

## 4. Conclusions

A series of PI composite films with POSS components in both the matrix and the fillers were designed and synthesized. This structural design reduced the AO erosion yields of the PI composite films. After an AO erosion with the fluence of 2.51 × 10^21^ atom/cm^2^, the composite films exhibited the erosion yield as low as 1.64 × 10^−26^ cm^3^/atom, which was ranked as one of the lowest values for the PI films reported in the literature. The good miscibility and compatibility of the POSS-substituted PI matrix and the POSS-containing additives achieved the molecular-level combination. This feature endowed composite films good uniformity, by which the active AO protection via DABA-POSS in the matrix and the passive AO protection via TSP-POSS in the fillers achieved good synergistic effects. The main disadvantage for the PI composite films might be the deteriorated high-temperature dimensional stability due to the internal plasticization effects of the POSS components. Fortunately, the relatively mild temperature cycle in LEO space environments might provide some practical applications for the composite films developed in this study. It is foreseeable that the excellent AO resistance of the POSS-containing PI composite films might provide long-term AO protection for LEO spacecraft in the future.

## Figures and Tables

**Table 1:** Formulation for the synthesis of POSS-PAAs.

PAA	PMDA (*M* = 218.12 g/mol)	ODA (*M* = 200.24 g/mol)	DABA-POSS (*M* = 1008.71 g/mol)	TSP-POSS (*M* = 931.34 g/mol)	DMAc (g)
POSS-PAA-10	20.00 g (91.69 mmol)	17.53 g (87.56 mmol)	4.17 g (4.13 mmol)	10.43 g	208.5 g
POSS-PAA-15	20.00 g (91.69 mmol)	17.06 g (85.21 mmol)	6.54 g (6.48 mmol)	10.90 g	218.0 g
POSS-PAA-20	20.00 g (91.69 mmol)	16.55 g (82.64 mmol)	9.14 g (9.06 mmol)	11.42 g	228.4 g
POSS-PAA-25	20.00 g (91.69 mmol)	15.98 g (79.80 mmol)	11.99 g (11.89 mmol)	11.99 g	239.9 g
POSS-PAA-30	20.00 g (91.69 mmol)	15.35 g (76.67 mmol)	15.15 g (15.02 mmol)	12.63 g	252.5 g

**Table 2:** Optical properties of POSS-PI composite films.

PI	*λ*_cut_^1^ (nm)	*T*_550_^2^ (%)	L* ^3^	a* ^3^	b* ^3^	Haze ^4^ (%)
POSS-PI-10	431	68.8	87.55	−6.88	83.04	7.19 (4.78)
POSS-PI-15	431	61.2	85.63	−4.02	87.99	16.63 (7.65)
POSS-PI-20	411	73.3	86.78	−5.52	82.75	11.79 (9.55)
POSS-PI-25	428	64.8	85.69	−4.08	86.26	14.15 (14.53)
POSS-PI-30	425	63.7	84.53	−2.41	87.73	15.82 (31.70)

^1^ Cutoff wavelength. ^2^ *T*_550_: Transmittance at the wavelength of 550 nm (thickness: 25 μm); ^3^ L*, a*, b*: color parameters calculated according to a CIE Lab equation. L* is the lightness, where 100 means white and 0 implies black. a*: positive value means red, negative value indicates green. b*: positive value means yellow, negative value indicates blue; ^4^ The data in the parentheses are the values without TSP-POSS additives [30].

**Table 3:** Thermal properties of POSS-PI composite films.

Samples	*T*_5%_^1^ (nm)	*T*_10%_^1^ (%)	*R*_w750_^1^ (%)	*T*_max_^1^ (°C)	CTE (×10^−6^/K)
POSS-PI-10	580 (534 ^3^)	608 (568)	70.6 (59.1)	624.6	61.0 (45.6 ^2^)
POSS-PI-15	572 (528)	601 (528)	70.7 (58.5)	620.7	74.7 (50.4)
POSS-PI-20	564 (524)	592 (548)	71.0 (59.6)	620.4	81.0 (56.1)
POSS-PI-25	559 (519)	585 (541)	71.3 (59.9)	611.9	79.6 (55.0)
POSS-PI-30	554 (512)	579 (531)	70.7 (59.3)	602.2	ND ^3^

^1^*T*_5%_: 5% weight loss temperature; *T*_10%_: weight loss temperature; *R*_w750_: residual weight ratio at 750 °C in nitrogen; *T*_max_: temperature at which the maximum thermal decomposition occurred. ^2^ The data in the parentheses are the values without TSP-POSS additives [30]. ^3^ Not detected.

**Table 4:** XPS results for the unexposed and exposed POSS-PI composite films.

Samples	*E*_s_^1^ (10^−26^ cm^3^/atom)	Relative Atomic Concentration (%)							
		Unexposed Samples				AO Exposed Samples			
		Si2p	C1s	O1s	N1s	Si2p	C1s	O1s	N1s
POSS-PI-10	9.21 (26.0 ^2^)	8.96	68.07	19.62	2.72	24.33	23.01	51.22	0.95
POSS-PI-15	6.06 (21.0)	4.90	74.65	16.63	2.94	23.36	27.56	47.78	0.70
POSS-PI-20	2.15 (16.9)	4.73	75.56	16.75	2.42	15.27	53.00	31.11	0.62
POSS-PI-25	1.82 (12.8)	4.94	74.93	16.81	2.87	21.33	30.77	46.20	1.45
POSS-PI-30	1.64 (11.1)	4.61	72.89	18.60	2.76	9.55	64.29	23.94	1.99
PI-ref ^3^	300	ND ^4^	ND	ND	ND	ND	ND	ND	ND

^1^ Erosion yield with the AO fluence of 2.51 × 10^21^ atom/cm^2^; ^2^ The data in the parentheses are the values without TSP-POSS additives with the AO fluence of 2.16 × 10^21^ atom/cm^2^ [30]; ^3^ PI_PMDA-ODA_; ^4^ Not detected.

## Data Availability

Data is contained within the article.
